# Development and validation of an enhanced PERCCI-S (PERCCI-S-Rev) for measuring person-centred home-based primary care

**DOI:** 10.3389/frhs.2026.1766556

**Published:** 2026-02-27

**Authors:** Theresa Larsen, Richard Sawatzky, Helle Wijk, Ewa Wikström, Axel Wolf

**Affiliations:** 1 The Gothenburg Region, Gothenburg, Sweden; 2 Institute of Health and Care Sciences, University of Gothenburg, Gothenburg, Sweden; 3 University of Gothenburg Centre for Person-Centred Care, Sahlgrenska Academy, Gothenburg, Sweden; 4 Centre for a Dementia Friendly Society with a Person-Centred Mindset, University of Gothenburg, Gothenburg, Sweden; 5 School of Nursing, Trinity Western University, Langley, BC, Canada; 6 Centre for Advancing Health Outcomes, Providence Health Care, Vancouver, BC, Canada; 7 Department of Architecture and Civil Engineering, Chalmers University of Technology, Gothenburg, Sweden; 8 Department of Quality Assurance, Sahlgrenska University Hospital, Gothenburg, Sweden; 9 Centre for Health Governance, Department of Management & Organization, The School of Business, Economics and Law, University of Gothenburg, Gothenburg, Sweden

**Keywords:** home-based primary care, item response theory, measurement evaluation, patient-reported experience measures, person-centred care, psychometrics

## Abstract

**Background:**

The Person-Centred Community Care Inventory – Swedish version (PERCCI-S) has been used in Swedish municipalities to measure person-centred care in home-based primary care (HBPC) from the patient's perspective since 2021. With use, areas for improvement have emerged. The aim was to develop a revised version that addresses issues identified in practice and incorporates recommendations from the previous validation study while maintaining or improving psychometric properties. A secondary aim was to confirm measurement equivalence of the PERCCI-S to support its use for patients with HBPC alone and those with both HBPC and social care services.

**Methods:**

Data were collected via two surveys of patients 18 years or older receiving municipal HBPC in Sweden including: a) 1 422 participants who completed the original PERCCI-S in 2023, and b) 1 204 who completed the revised version in 2024. The revised PERCCI-S was developed based on prior feedback and validated based on a series of psychometric and item-response theory analyses comparing the two versions.

**Results:**

The revised PERCCI-S consists of 12 items. One item was replaced, and three were reworded to emphasize partnership, autonomy, and emotional attunement, core elements of contemporary person-centred care frameworks. Additionally, three items were reworded for clarity. The response scale was changed from four to seven response options. Results of psychometric analyses provide support for the measurement structure of the revised PERCCI-S and measurement equivalence between patients with HBPC alone and those with both HBPC and social care services. The overall scale has good internal consistency reliability (*α* = 0.97) and fewer ceiling effects compared to the original.

**Discussion:**

The revised PERCCI-S (PERCCI-S-Rev) improves on the original with clearer wording and expanded response options that better distinguish patients’ experiences, while maintaining strong psychometric properties.

## What is already known about the topic?

- Despite the global paradigm shift towards more health care being provided outside hospitals, few validated instruments measure patient's experience of person-centred care for use in home-based settings.
- The Person-Centred Community Care Inventory – Swedish version (PERCCI-S) has been used in Swedish municipalities to measure person-centred care in home-based primary care from the patient's perspective since 2021.

## What does this paper add?

- Reports the development and psychometric evaluation of the revised version (PERCCI-S-Rev).
- Introduces PERCCI-S-Rev as a 12-item self-report questionnaire with improved psychometric properties for assessing person-centredness in home-based primary care from the patient's perspective.
- Shows measurement equivalence of the PERCCI-S-Rev when comparing patients receiving only home-based primary care and those also receiving social services.

## Implications for practice, theory, or policy

- PERCCI-S-Rev can be used in studies to describe person-centredness in home-based primary care from the patient's perspective. For quality improvement purposes, the PERCCI-S-Rev can be used to identify areas where person-centredness in home-based primary care can be improved.
- PERCCI-S-Rev's usefulness in other populations and for evaluating the impact of interventions and services aimed at improving person-centredness for patients receiving home-based primary care remains to be tested.

## 1 Background

Globally, health care systems are undergoing significant transformation, shifting towards integrated care approaches that prioritize delivering services in more flexible environments, including patients' homes, rather than traditional hospital or institutional settings (1–3). This shift underscores the importance of designing health and social care systems that are centred on the basis of individual needs of patients rather than being dictated by the requirements and structures of organisations (4–6).

Achieving and maintaining person-centred care (PCC) requires reliable tools to measure how well the objectives of PCC are being met (7). Although numerous instruments exist to assess PCC (8), most were developed for institutional settings and therefore do not capture the relational, longitudinal, and often fragmented nature of home-based primary care (HBPC) (9, 10). This gap became evident in Sweden in 2020 when no validated patient-reported instrument in Swedish existed for HBPC. In response, a patient-reported experience measure (PREM) tailored for HBPC within the Swedish municipal health care system was developed and tested (PERCCI-S) (11), which was based on the 12-item version of the Person Centred Community Care Inventory (PERCCI) (12). Importantly, the PERCCI-S was developed in the context of a quality improvement project, with the aim of creating a tool for evaluating person-centredness in routine care.

The PERCCI was originally created in English through a participatory process involving older adults living in the community and receiving a mix of mental health and social care services in Great Britain. The final version of the questionnaire consisted of 18 items and demonstrated strong validity and reliability (13). In 2020, a condensed 12-item version of the PERCCI was made available online but has not yet undergone validation (12).

While social care constituted an explicit domain in the original UK context, the Swedish adaptation deliberately focused on experiences of municipal home-based primary health care, reflecting differences in service organisation and scope. Nevertheless, many patients receiving HBPC also receive social care services, which has implications for how items may be interpreted and underscores the need for careful evaluation of measurement equivalence across patient groups.

The adaptation process therefore focused on retaining items that captured relational and interactional aspects of person-centred care—such as being listened to, respected, and involved in decisions—while ensuring that item wording was explicitly anchored in experiences of municipal HBPC rather than social care or mental health services. For example, items were reworded to emphasise professional trust rather than personal closeness, to specify health- and care-related communication, and to replace community participation—which is the responsibility of social care in Sweden—with care coordination, a core component of person-centred HBPC and integrated care. Relevance and content validity in the Swedish context were secured through a mixed-method process including forward–backward translation, focus groups with municipal health-care professionals and managers, cognitive interviews with patients receiving HBPC, and expert review, as described in the previous evaluation of the PERCCI-S (11).

### 1.1 The original PERCCI-S

The PERCCI-S has been used since 2021 to measure person-centredness from the patient's perspective in HBPC in 12 Swedish municipalities in western Sweden as part of the Gothenburg key figures on integrated municipal primary care (14, 15). Its content and measurement properties have been evaluated using a mixed-methods approach involving qualitative analyses and item response theory (11). Although the PERCCI-S demonstrated acceptable psychometric performance, subsequent use of the PERCCI-S revealed areas where clarity, conceptual alignment, and discriminatory ability could be strengthened. In particular, some items could be reworded to more explicitly reflect contemporary perspectives on person-centred care that emphasise partnership between caregivers and patients (11, 16).

In addition, the original four-point response scale showed limited discrimination, with pronounced ceiling effects (11). Methodological research suggests that reliability and discriminating power increase as the number of response categories increases from very few options, with gains levelling off at around seven categories while respondent burden increases with longer scales (17–19). For bipolar agreement items such as those used in the PERCCI-S, guidance recommends up to seven response categories (20). Accordingly, a seven-point response scale was selected to address the identified measurement limitations while balancing psychometric performance and feasibility for a predominantly older HBPC population.

Cognitive interviews with patients conducted during the original validation also highlighted patient concerns related to continuity of care and accessibility, including experiences of limited continuity and difficulties contacting HBPC when needed (11). These findings raised questions about whether such aspects represent core components of the same latent construct of person-centredness or reflect related but conceptually distinct features of care delivery.

Empirical evaluations of the PERCCI-S have consistently demonstrated a dominant unidimensional structure, supporting its use as an overall measure of patients perceived person-centredness in municipal HBPC. Retaining a unidimensional structure offers important advantages for interpretability, scoring, routine quality improvement and benchmarking. Accordingly, the PERCCI-S is conceptualised as a reflective measure, in which individual items are understood as manifestations of a latent construct representing patients' experiences of person-centred HBPC; under this conceptualisation, internal consistency and structural validity (e.g., of a unidimensional structure) are relevant measurement properties, consistent with COSMIN guidance (21). The present study therefore evaluated whether aspects related to continuity and accessibility could be incorporated without compromising this unidimensional structure, while recognising that such aspects may be better captured as complementary indicators or separate dimensions in future research.

### 1.2 Primary care and homecare in Sweden

In Sweden, primary care is provided by both regional and municipal services. Municipal health care covers care and treatment in nursing homes, day care facilities, and home health care in ordinary housing, but does not include medical care delivered by physicians (22). In 2024, more than 400 000 people received municipal health care in Sweden (23). The main professional groups working in municipal health care are registered nurses and occupational therapists. Much of the practical care is delegated to assistant nurses, care assistants, and staff without formal medical training, primarily employed in social services and nursing homes (i.e., not within the same organisation as the municipal primary health care). Typical interventions include wound care, injections, catheter management, and provision of mobility aids such as wheelchairs and walkers, but more advanced procedures like peritoneal dialysis, blood transfusions, and intravenous antibiotics are also performed (22, 24).

Approximately 70% of individuals receiving municipal primary care also receive support from social services with non-medical tasks such as cleaning, cooking, and personal hygiene. Evidence from previous studies indicates that effective cooperation between social services and HBPC is important for the HBPC to be experienced as person-centred (25, 26). Evidence also suggests that people, on average, are more satisfied with HBPC than with social services (25, 27).

In the municipalities where the PERCCI-S has been used, there has been an ongoing discussion regarding how to interpret the results of the PERCCI-S when the sample includes those who only received HBPC and others who also received social services. Some stakeholders have argued that differences in total PERCCI-S scores could be confounded by patients’ satisfaction with social services. Although social care is not within the intended measurement scope of the PERCCI-S, the high prevalence of co-receipt of social care among patients receiving municipal HBPC means that experiences of social care may still influence how patients interpret and respond to questionnaire items. To ensure comparability of the PERCCI-S scores across these care contexts, it is therefore important to confirm measurement equivalence between patients receiving HBPC alone and those receiving HBPC in combination with social care services.

### 1.3 The present study

This study is part of ongoing efforts to evaluate and refine the PERCCI-S. The aim was to develop a revised version (PERCCI-S-Rev) that addresses issues identified in practice and incorporates recommendations from the previous validation study while maintaining or improving psychometric properties. Our corresponding analytical objectives were to examine whether (1) reworded or replaced items demonstrated improved psychometric discrimination and information, improved convergent validity, and reduced missing responses, (2) more response options were associated with increased information and reduced ceiling effects, and (3) new items contributed to improved psychometric information and reduced missing responses, and was compatible with retaining a unidimensional measurement model (Table 1). A secondary aim (and fourth analytical objective) was to confirm measurement equivalence of the PERCCI-S-Rev to support its use for patients with HBPC alone and those who received both HBPC and social care services.

**Table 1:** Analytical objectives and decision framework for evaluating revisions to the PERCCI-S.

Analytical objectives	Analyses conducted	Decision guidelines^a^
1. Evaluate whether reworded or replaced items demonstrate improved psychometric discrimination and information, and reduced missing responses relative to the original PERCCI-S.	Each reworded or replaced item in the revised PERCCI-S (Sample B, 2024) was evaluated relative to the corresponding item in the original PERCCI-S (Sample A, 2023) by: a) conducting item-response theory (IRT) analyses to graphically compare improvements in item discrimination information b) comparing the portion of missing responses for each item in the original and revised version.	A reworded or replaced item was retained if: a) The standardized factor loading was greater than 0.65, and the relative improvement in discrimination and information for the reworded or replaced item exceeded that observed for non-reworded items b) The percentage of missing responses was smaller for reworded or replaced items.
2. Evaluate whether the addition of one or two new items could be accommodated within a unidimensional PERCCI-S without loss of psychometric performance.	Each new item was evaluated by conducting IRT analyses to investigate item discrimination.	A new item was retained if: a) The standardized loading was greater than 0.65. b) There was no loss in model fit compared to the model with 12 items.
3. Examine whether the revised response scale was associated with increased measurement information and reduced ceiling effects relative to the original PERCCI-S.	Total score distributions and information functions were compared between the original PERCCI-S (Sample A, 2023) and the revised PERCCI-S-Rev (Sample B, 2024) by: a) conducting IRT analyses to compare item category characteristic curves and total information curves, and b) calculating and visually inspecting total score distributions.	The revised response scale was considered to perform favourably if: a) item category characteristic curves and total information curves indicated improved spread across the latent continuum, and b) fewer than 15% of respondents attained the highest possible total score.
4. Determine measurement equivalence of the PERCCI-S-Rev across patients receiving HBPC alone versus HBPC in combination with social care services.	Multi-group measurement invariance analyses to test for configural, metric, and scalar invariance.	Measurement equivalence was supported when the sequential measurement invariance tests did not result in a statistically significant decrement in model fit.

a Not all decision guidelines need to be met for an item to be retained. Psychometric properties of the original PERCCI-S are reported in detail elsewhere (11) and are included here for comparative reference only.

## 2 Methods

### 2.1 Study design, population and data collection

Data were obtained via postal questionnaires administered to two cross-sectional samples of patients receiving HBPC in western Sweden in two consecutive years. Sample A (2023) completed the original PERCCI-S and has been reported previously (11), whereas Sample B (2024) completed the revised version of the instrument (PERCCI-S-Rev) and is reported for the first time in the present study. The two samples were used to examine differences in measurement properties between the original and revised versions of the scale.

Sample A comprised patients aged 18 years or older receiving HBPC in October 2023. The questionnaire was distributed to all eligible patients in eight municipalities (*n* = 1 486). In addition, a random sample of 1 512 eligible patients was drawn from four other municipalities, resulting in a total sample size of 2 988.

Sample B comprised patients aged 18 years or older receiving HBPC in September 2024. The questionnaire was distributed to all eligible patients in ten municipalities (*n* = 2 145). In addition, a random sample of 944 eligible patients was drawn from two other municipalities, resulting in a total sample size of 3 089.

The same municipalities participated in both the 2023 and 2024 surveys. Eligibility criteria and data collection procedures were identical in both years (patients aged ≥18 years receiving municipal HBPC during the survey month; identical survey administration protocol). However, the sampling strategy varied by municipality and year. These differences reflect municipality-specific sampling decisions rather than differences in target populations, as the underlying eligibility definition was constant across municipalities and years. Two municipalities that used random sampling in 2023 invited all eligible patients in 2024, as the difference between random sampling and total inclusion in these municipalities was small (approximately 200 patients). These differences were therefore unlikely to substantially influence the results, particularly given the similarity of respondent characteristics across samples (Table 2).

**Table 2:** Respondent characteristics by survey year (sample A, 2023; sample B, 2024) and test of differences between cohorts.

Respondent characteristics	Sample A (2023) *n* = 1 422 n (%)	Sample B (2024) *n* = 1 204 n (%)	Test statistic (df)	*p*-value
Age groups	*n* = 1 334	*n* = 1 155	U = 802005.5 Z = 1.85	0.06
Under 65	125 (9.4)	99 (8.6)		
65–69	89 (6.7)	55 (4.8)		
70–79	317 (23.8)	256 (22.2)		
80–89	511 (38.3)	483 (41.8)		
90 or over	292 (21.9)	262 (22.7)		
Sex	*n* = 1 372	*n* = 1 163	*χ*^2^ (1) = 2.11	0.15
Men	770 (56.0)	686 (59.0)		
Women	602 (43.8)	477 (41.0)		
Cohabitation	*n* = 1 374	*n* = 1 158	χ^2^ (1) = 0.44	0.51
Yes	475 (34.6)	415 (35.8)		
No	899 (65.4)	743 (64.1)		
Overall health	*n* = 1 372	*n* = 1 163	U = 779 454.5 Z = −1.05	0.29
Very good	76 (5.6)	68 (5.8)		
Fairly good	379 (27.6)	246 (21.2)		
Fair	568 (41.4)	474 (40.8)		
Fairly poor	290 (21.1)	321 (27.6)		
Poor	59 (4.3)	54 (4.6)		
Problems with worry or anxiety	*n* = 1 364	*n* = 1 156	U = 786 203.5 Z = −0.13	0.89
No	679 (49.89	584 (50.5)		
Yes, somewhat	560 (41.1)	457 (39.5)		
Yes, severely	125 (9.2)	115 (9.9)		
Receipt of social care service	*n* = 1 371	*n* = 1 155	χ^2^ (2) = 8.99	0.003
Yes	988 (72.19)	890 (77.1)		
No	328 (23.99)	220 (19.0)		
Don't know	55 (4.09)	45 (3.9)		
Care frequency	*n* = 1 363	*n* = 1 143	U = 711 142.0 Z = −0.90	0.37
3 or more times per day	675 (49.6)	589 (51.5)		
1–2 times a day	325 (23.89)	261 (22.8)		
More than once a week	147 (10.89)	107 (9.4)		
Once a week	74 (5.49)	70 (6.1)		
Less often than once a week	95 (7.09)	76 (6.6)		
Don't know	47 (3.5)	40 (3.5)		
Country of birth	N.A	*n* = 1 124		
Sweden		995 (88.1)		
Nordic countries other than Sweden		53 (4.9)		
Europe other than Nordic countries		44 (4.1)		
Outside Europe		32 (3.0)		
Highest level of education	N.A	*n* = 1 047		
Primary school		436 (41.6)		
High school		320 (30.6)		
University		291 (27.8)		
Assistance with questionnaire completion	*n* = 1 358	*n* = 1 204	χ^2^ (1) = 1.75	0.19
Yes	752 (55.4)	699 (58.0)		
No	606 (44.6)	505 (42.0)		
Modality of questionnaire	*n* = 1 429	*n* = 1 204	χ^2^ (1) = 0.37	0.54
Paper	1 308 (92.0)	1 131 (93.4)		
Digital	114 (8.0)	80 (6.6)		

N.A, not available. Country of birth and highest level of education were collected only in the 2024 survey (Sample B) and were therefore not included in between-cohort comparisons. Pearson's Chi-Square tests were used to compare the distribution of sex, cohabitation, receipt of social care services, help completing the questionnaire and modality of questionnaire. The Mann–Whitney U test was used to compare age groups, overall health, problems with worry or anxiety and home care frequency.

All surveys included one item on overall satisfaction with municipal HBPC and background questions on age, gender, overall health, problems with worry or anxiety, cohabitation, and whether someone assisted in completing the survey. We also asked the patients about frequency of HBPC and whether patients received social care services (because we lacked data from municipal registers). In addition, the 2024 survey (sample B) included questions on place of birth and highest level of education.

Respondents were explicitly instructed to base their responses on the care they received from the HBPC, including registered nurses, occupational therapists, physiotherapists, assistant nurses, and care assistants. Examples of relevant HBPC services were provided (e.g., medication management, wound care, blood sampling, rehabilitation, and provision of assistive devices). Respondents were also instructed not to base their response on experiences with social care services (e.g., assistance with personal hygiene, meals, or cleaning) or physician-provided medical care. When questionnaires were completed by a relative, respondents were instructed to answer based on their perception of the patient's experiences, rather than their own. All items referred to experiences during the preceding month.

Patients could choose to answer a digital version of the questionnaire or a paper version and post it in a prepaid envelope. Every patient was given an identification code, and the code was printed on the questionnaire. This made it possible to identify which patients had replied to the questionnaire. One reminder was sent. Only a contact person in each participating municipality had access to the code key. Each contact person was required to sign a protocol certifying that they had followed the procedure for conducting the patient survey as outlined by the researchers in the project.

### 2.2 Analysis

Various statistical approaches with corresponding decision guidelines were used iteratively to determine whether to a) retain any of the wording changes to nine of the items, b) add two new items, and c) change the response scale from four to seven response option (Table 1). Our guiding principle was to retain the original items unless there was compelling evidence to do otherwise.

Because multiple changes were introduced simultaneously between survey waves—including item wording, response categories, and the addition of new items—the present design did not permit isolation of the effects of individual modifications. Accordingly, the analyses focused on the comparative psychometric performance of the revised instrument rather than on estimating causal effects attributable to individual modifications. Comparisons between the original and revised versions are intended to provide contextual benchmarking rather than causal inference.

Patient characteristics, item and total score distributions and missing data patterns were assessed using descriptive statistics. Total-score distributions were evaluated for floor and ceiling effects, defined as occurring when over 15% of respondents attain the lowest or highest possible scores (28). Total score distributions were calculated by summing the item responses for each participants. To visualise and compare score distributions across versions, raw total scores were rescaled to a 0–100 metric. A histogram-style binned-statistics approach was then applied: scores were grouped into 10-point intervals (e.g., 0–10, 10–20, etc.), and the proportion of respondents within each interval was calculated. Proportions were used rather than raw counts to enable direct comparison between instruments with different response formats.

Given our aim to evaluate the proposed revisions to the PERCCI-S (sample B) relative to the original PERCCI-S (sample A), it was important to first assess whether demographic as well as care and health-related characteristics are comparable for both samples. Pearson's Chi-Square tests were conducted to compare the distribution of sex, cohabitation, respondents also having social services and those who had assistance completing the questionnaire. The Mann–Whitney U test was used to compare ordinal variables across both samples, including age groups, overall health, problems with worry or anxiety and home care frequency. For age, respondents were grouped into five categories: 18–65, 65–74, 75–84, 85–89, and 90 years or older. Overall health was measured on a five-point ordinal scale ranging from *very poor* to *very good*. Problems with anxiety was measured on a three-point ordinal scale with the response options *No*, *Somewhat* and *Severely*. Care frequency was measured on a five-point ordinal scale with the response options *Three or more times a day*, *Once or twice per day*, *Several times a week*, *Once a week* and *Less often than once a week*. All tests were two-tailed, and statistical significance was set at *p* < 0.05.

To assess the psychometric characteristics of the two new and nine reworded or replaced items, we conducted item response theory (IRT) analyses. As a preliminary step, we first conducted an exploratory factor analysis (EFA) to determine whether the unidimensional structure of the PERCCI-S was retained following item revisions (Sample B). The weighted least squares mean- and variance-adjusted (WLSMV) estimator was used to accommodate the ordinal items. The number of factors was determined based on a combination of statistical information (eigenvalues, factor loadings, and model fit indices) and clinical considerations. Model fit was evaluated using multiple indices, including the comparative fit index (CFI) and Tucker–Lewis index (TLI), with values ≥0.95 indicating good fit, and the standardized root mean square residual (SRMR), with values >0.10 indicating the possibility of poor model fit (29). Because large samples can inflate chi-square statistics, greater emphasis was placed on factor dominance, patterns of residuals, and the interpretability of the factor solution when evaluating dimensionality.

Subsequently, Samejima's 2-parameter graded response model was fit to the data to graphically compare item characteristics and information curves, and total information resulting from each revision, relative to the original PERCCI-S (30, 31). We observed whether the item characteristic curves and information curves of the reworded or replaced items relative to the original items demonstrated improved monotonicity (ordered item distributions, improved discrimination, and greater spread (or coverage) across a wider range of the latent trait (theta). We further observed whether any improvements for the reworded or replaced items were greater than those for non-reworded items. Statistical tests comparing item discrimination and information across both versions could not be conducted because the rewording or replacement of items and the addition of new items could not be isolated from the increased number of response options (which applied to all items). New, replaced or reworded items were only retained if the standardized discrimination factor loadings (discrimination) were greater than 0.65 as suggested by Baker (32). Additionally, new items were only retained if there was no loss in model fit compared to the original PERCCI-S (based on the standardized bivariate residuals for all item pairs) (33).

Measurement equivalence of the revised PERCCI-S between patients receiving social services or not was evaluated by conducting series of multi-group measurement invariance analyses (34). The final IRT model of the revised PERCCI-S was used as the baseline model. First, a configural model was specified in which the overall measurement structure was held constant across groups. Next, a metric invariance model was tested by constraining item discrimination parameters (factor loadings) to equality, followed by a scalar invariance model that also constrained item threshold parameters. Model comparisons were based on chi-square difference testing using the MLR estimator. Measurement invariance was considered supported when more constrained models did not show a statistically significant (*p* < 0.05) decrement in model fit relative to the less constrained model.

Missing data were addressed in the analyses following well-established recommendations (35). Full information maximum likelihood was used to accommodate missing item responses in all IRT analyses, and pairwise deletion was used for the EFAs, thus allowing for the use of available item responses under the missing at random assumption. Multiple imputation was used to accommodate item-level missing data for analyses of total scores.

Potential covariates of missingness were identified by conducting univariable logistic regression analyses with incomplete response as the outcome and respondent characteristics (age, sex, overall health, problems with worry or anxiety, cohabitation, frequency of HBPC, receipt of social services, and assistance with survey completion) as predictors. Response completeness was dichotomised as complete (all 12 items answered) or incomplete (one or more items missing). Statistically significant covariates (*p* < 0.05) were subsequently included as auxiliary variables for creating 10 multiple imputation datasets.

The descriptive statistics (e.g., respondent characteristics and distribution of item categories) were calculated in IBM SPSS, version 29.0.0.1. Most latent variable analyses (IRT, EFA, and measurement invariance analyses) and multiple imputations were carried out in Mplus software, version 8.10 (36).

### 2.3 Ethical considerations

This study was conducted according to the principles of the 1996 Declaration of Helsinki (37). Ethics approval for the study was obtained from the Swedish Ethical Review Authority (Dnr 2023-04968-01, 2023-04968-02 and 2024-04425-01). Participation was voluntary, and participants could withdraw from the study at any time. All participants received written information about the study and gave consent to participate by submitting the questionnaire.

We distributed the questionnaire by post to maximize response rates and ensure accessibility. Most patients receiving HBPC in Sweden are aged 80 years or older (38). Although digital competence is increasing among older adults, not everyone has the skills or devices required to complete an online survey. Given the high prevalence of multimorbidity and cognitive difficulties in this population, respondents were encouraged to complete the questionnaire with support from a relative or another person. If the patient was unable to participate, relatives were invited to respond on their behalf, instructed to answer from the patient's perspective rather than their own. Despite these efforts, the response rate was likely lower among those with the greatest care needs, poorer health, or limited proficiency in Swedish, as the survey was only available in that language.

## 3 Results

To aid interpretation, results comparing the original PERCCI-S (Sample A) and the revised version (Sample B) are presented to contextualize the psychometric performance of the revised instrument relative to the original and should not be interpreted as estimates of causal effects.

### 3.1 Participants

After one reminder, the response rate of the questionnaire was 48% (*n* = 1 422) in 2023 (sample A, using the original PERCCI-S) and 39% (*n* = 1 204) in 2024 (sample B, using the revised PERCCI-S). Only respondents who had replied to at least one PERCCI-S item were included in the sample (seven respondents were excluded from each sample because of not having replied to any of the PERCCI-S items but having replied to other questions in the questionnaire). Patients who passed away during the survey period (1%–10% of the population depending on the municipality and year) were excluded from the denominator for calculating response rates.

Samples A and B were similar regarding their distributions of age, sex, cohabitation, overall health, problems with anxiety, care frequency or if someone assisted with completing the questionnaire. Observed differences in the results of the two versions of PERCCI-S are therefore unlikely to be explained by these differences in respondent characteristics. However, the proportion of respondents who besides HBPC also had social services was significantly higher in 2024 compared to 2023 (77.1% vs. 72.1%). Further details of both samples are given in Table 2.

**Figure 1:**
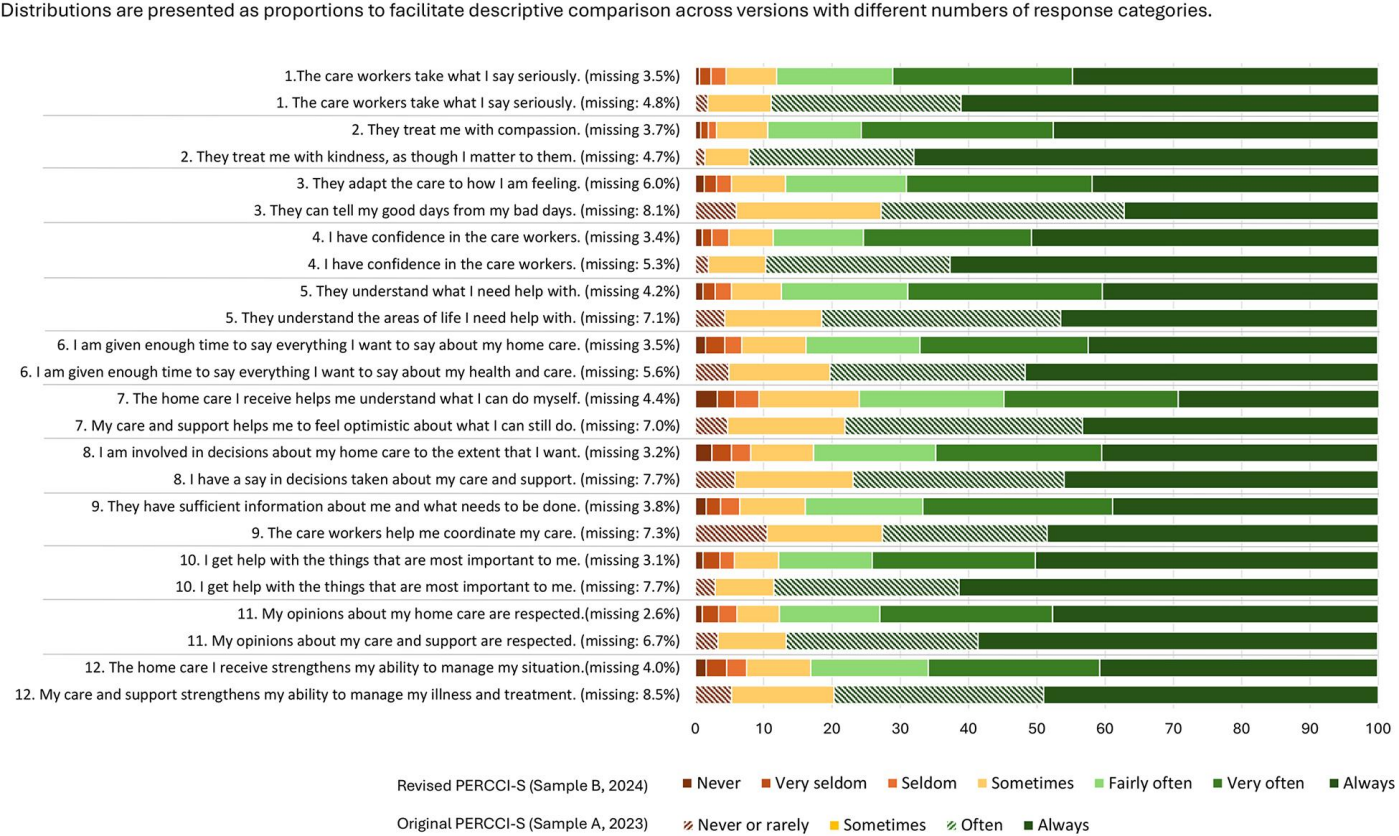
Relative frequency distribution of selected response options and missing responses for each item in the revised PERCCI-S (sample B, 2024) and the original (sample A, 2023).

The frequency distributions and percentages of missing responses for each item of the PERCCI-S (sample A) and the revised PERCCI-S (sample B) are presented in Figure 1. In total, only 3.2% of all possible responses were missing across the 12 items. The percentage of item-level missing data was lower for all items for the revised PERCCI-S compared to the original, ranging from 2.6% (item 11: My opinions about my home care are respected) to 6.0% (item 3: They adapt the care to how I am feeling). As a comparison, item-level missing data for the original version ranged from 4.5% (item 1: The care workers take what I say seriously) to 8.2% (item 3: They can tell my good days from my bad days). A total of 165 respondents (13.6%) did not respond to one or more items on the revised version (sample B) compared to 18.3% (*n* = 261) for the original version (sample A). For the revised version (sample B) a total of 77 respondents (6.8%) did not respond to one of the items (sample A: 7,5%), 20 respondents (1.7%) did not respond to two of the items (sample A: 2.7%), and 7 (0.6%) (sample A: 2.6%) did not respond to any item but did respond to one or more of the other questions in the questionnaire.

### 3.2 Patterns of item nonresponse

In univariable analyses, higher age, fewer problems with worry or anxiety, and lack of assistance when completing the questionnaire were associated with higher odds of incomplete response (Table 3). Other respondent characteristics were not significantly associated with incomplete response.

**Table 3:** Univariable associations between respondent characteristics and incomplete questionnaire response (one or more PERCCI-S items missing).

Variable	n	OR	95% CI	*p*-value
Age group (ordinal)	1 155	1.13	1.03–1.25	.012
Sex	1 163	0.92	0.74–1.14	.451
Cohabitation	1 158	1.20	0.95–1.50	.128
Overall health	1 163	1.06	0.95–1.19	.307
Problems with worry or anxiety	1 156	1.27	1.07–1.51	.006
Receipt of social services	1 155	1.06	0.81–1.38	.683
Care frequency	1 143	1.04	0.95–1.14	.406
Assistance with questionnaire completion	1 204	0.69	0.56–0.86	<.001

Odds ratios (OR) estimated using univariable binary logistic regression. For ordinal variables, odds ratios represent the change in odds of incomplete response per one-category increase. Sample sizes (n) vary due to item-level nonresponse in the covariates.

### 3.3 Evaluating the reworded items

The EFA indicated a dominant general factor underlying the revised PERCCI-S, just as for the original PERCCI-S (11). The first and second eigenvalues were 9.28 and 0.69 (a ratio of 13.4), and the unidimensional model resulted in high comparative fit index values (CFI = 0.98; TLI = 0.97) and low residuals (SRMR = 0.04).

All items of the revised PERCCI-S demonstrated substantial standardized factor loadings based on the IRT model; the loadings of the PERCCI-S with reworded items ranged from 0.78 to 0.93, thus exceeding the commonly accepted threshold of 0.65 (Table 4). When compared to the IRT results of the original version, standardized factor loadings increased for nearly all of the other items (1–12), strengthening the evidence for improved construct representation in the revised PERCCI-S. The exceptions were item 7 (.78 vs..83) and item 12 (.86 vs..88), where slight decreases were observed.

**Table 4:** Standardized factor loadings for alternative PERCCI-S model specifications across survey samples.

Item	Model 1 Original PERCCI-S	Model 2 PERCCI-S with reworded items	Model 3 PERCCI-S with new and reworded items
	Standardized loading [95% CI]	Standardized loading [95% CI]	Standardized loading [95% CI]
1	.86 [.84-.89]	.87 [.85-.89]	.87 [.85-.90]
2	.86 [.83-.88]	.88 [.86-.90]	.88 [.85-.90]
3	.73 [.70-.77]	.89 [.87-.91]	.89 [.87-.91]
4	.85 [.83-.89]	.90 [.88-.92]	.90 [.88-.92]
5	.84 [.83-.88]	.90 [.89-.92]	.90 [.89-.92]
6	.84 [.81-.87]	.89 [.87-.91]	.89 [.87-.91]
7	.83 [.80-.87]	.78 [.75-.81]	.78 [.75-.81]
8	.80 [.78-.84]	.86 [.84-.88]	.86 [.84-.89]
9	.79 [.76-.82]	.86 [.83-.88]	.86 [.83-.88]
10	.90 [.88-.92]	.91 [.89-.92]	.91 [.89-.93]
11	.92 [.91-.94]	.93 [.92-.94]	.93 [.91-.94]
12	.88 [.85-.90]	.86 [.84-.88]	.86 [.84-.89]
13			.40 [.34-.46]
14			.43 [.37-.50]

Model 1 is based on the original PERCCI-S administered in 2023 (Sample A), whereas Models 2 and 3 are based on the revised questionnaire administered in 2024 (Sample B). Factor loadings for Model 1 have been reported previously (11) and are reproduced here for comparative reference. Model 1 includes 1 422 respondents and Models 2 and 3 include 1 204 respondents (seven respondents in each sample were excluded due to no responses to any PERCCI-S items). All models were estimated using full-information maximum likelihood (FIML). Differences in factor loadings across models should be interpreted descriptively and reflect differences in item wording and survey samples rather than causal effects of specific modifications.

**Figure 2:**
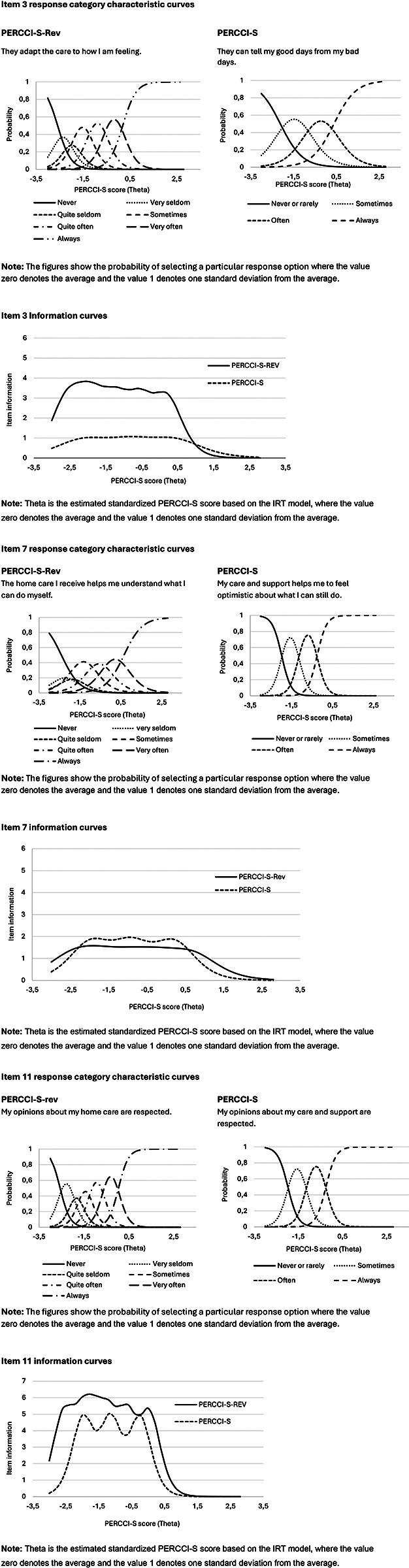
Comparison of response category characteristic curves and information curves for three items in the revised and original version of PERCC-S.

The IRT information curves indicate greater statistical information for all items of the revised PERCCI-S compared to the original, except for item 7 (all item category characteristic and information curves can be found in the Supplementary Material). Figure 2 illustrates how the revised wording affects item functioning across the response continuum, using representative items with the highest, average and lowest information values. For example, Item 11 (revised version: “My opinions about my home care are respected”, original version: “My opinions about my care and support are respected”) provides the highest level of statistical information compared to all other items in both the original and revised version. However, this information is mostly focused on individuals with lower scores on the PERCCI-S scale. In contrast, Item 7 (revised version: “The home care I receive helps me understand what I can do myself”, original version: “My care and support helps me to be optimistic about what I can still do”) provides the least amount of information in the revised version but offers the most statistical information for individuals with higher scores on the scale. Item 3 (revised version: “They adapt the care to how I feel”, original version: “They can tell my good days from my bad days”) provides the least amount of information in the original version but provides a moderate level of information in the revised version.

### 3.4 Evaluating the new items

The IRT analysis indicates that the two new items had lower factor loadings below the commonly accepted threshold of 0.65 and resulted in worse model fit when compared to the revised PERCCI-S with only the replaced or reworded items (i.e., statistically significant standardized bivariate residuals increased to 15.3% from 14.5%, and 21.5% of the standardized residuals for the new items were statistically significant.

### 3.5 Evaluating the number of response options

**Figure 3:**
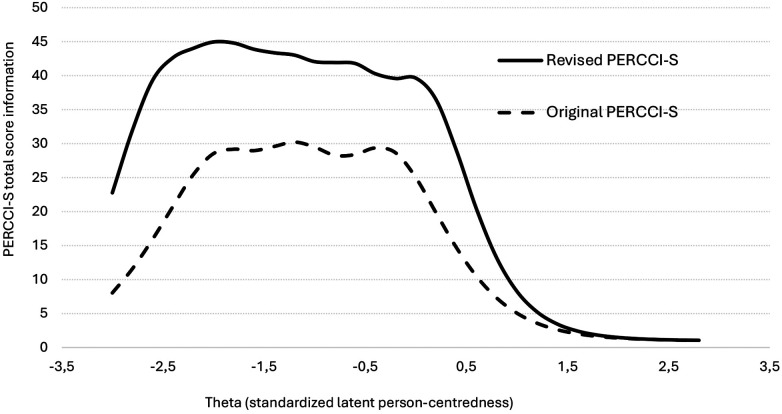
Total information curves for the original PERCCI-S (Sample A, 2023) and the revised PERCCI-S-Rev (Sample B, 2024). Note: Theta is the estimated standardized PERCCI-S score based on the IRT model, where the value zero denotes the average and the value 1 denotes one standard deviation from the average. Lower values indicate less person-centred care. Differences between curves should be interpreted descriptively and reflect differences in instrument version and survey samples rather than causal effects of specific modifications.

The IRT total information curve showed that the revised version of the PERCCI-S, which had seven response options, compared to the original four, provides more statistical information (i.e., greater reliability) across a wider range of scores, compared to the original (Figure 3). The improved information of the revised PERCCI-S is particularly noteworthy for lower theta scores, indicating that greater reliability is achieved for those with lower and average PERCCI-S scores.

The overall internal consistency reliability (ordinal alpha) of the original PERCCI-S was 0.97 and 0.96 for data set A and B, respectively, based on unidimensional CFAs. For the revised version informed by IRT analysis, the ordinal alpha was 0.98, indicating excellent internal consistency.

**Figure 4:**
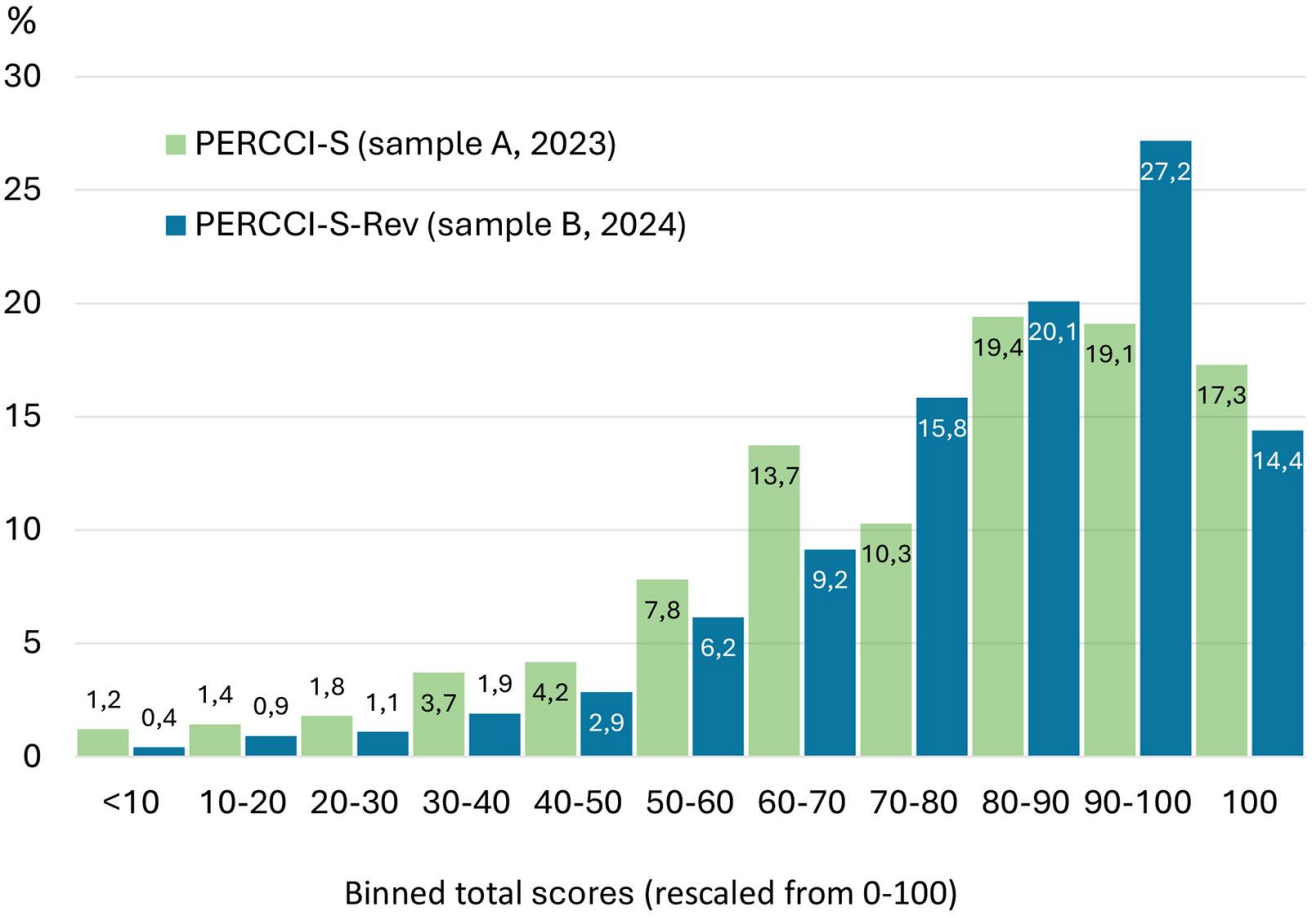
Distribution of binned total scores for the original PERCCI-S (sample A, 2023) and the revised version PERCCI-S-Rev (sample B, 2024). Note: The PERCCI-S total score distributions were calculated based on missing values replaced by 10 multiple imputations. Lower values indicate lower person-centred care. Differences between distributions should be interpreted descriptively and reflect differences in instrument version and survey samples rather than causal effects of specific modifications.

Compared to the original PERRCI-S, the revised version demonstrated a modest improvement at the top end of the scale, with fewer respondents achieving the maximum score. The original PERCCI-S had a ceiling effect of 17.3% of the respondents scoring the highest possible score (based on 10 multiple imputations), whereas ceiling effect was reduced to 14.4% for the revised PERCCI-S, which falls just below the suggested threshold of ≥15% (28). While high scores continued to cluster in the 90%–100% range, the reduced proportion of maximum scores indicates improved discrimination among respondents scoring at the upper extreme (Figure 4).

### 3.6 Evaluating measurement equivalence

Measurement invariance of the revised PERCCI-S was evaluated between patients receiving only HBPC (*n* = 218) and those also receiving additional social care services (*n* = 886) (45 respondents were excluded because they replied that they did not know, 49 were excluded because they did not reply to the question about social services and 6 were excluded due to non-response for all of the PERCCI-S items). Multi-group IRT analysis indicated that both metric invariance [*Δχ*^2^ (11) = 11.98, *p* = .365] and scalar invariance [*Δχ*^2^ (82) = 76.31, *p* = .657] were supported. These results demonstrate that the revised PERCCI-S has an equivalent unidimensional measurement structure and measurement properties across the two patient groups, allowing for meaningful comparisons of the revised PERCCI-S scores across both groups.

### 3.7 The final revised version of PERCCI-S

Comparisons of reworded items with their original versions (see Objective 1 in Table 1) showed that most revisions improved the psychometric properties of the scale, as indicated by higher IRT factor loadings and more information being provided in item response category characteristic and information curves, with the exception of item 7 and 12. Accordingly, the revised wordings were adopted for all items except items 7 and 12, where the original wording was retained. Replacing the original item 9 with a newly developed item also resulted in better discrimination (larger standardized loadings), and the new item was therefore included in the final version. In contrast, although care continuity and care access are recognized as central elements of person-centred care, the two new items addressing these aspects did not yield psychometric improvements and were consequently excluded from the final revised PERCCI-S (PERCCI-S-Rev). The results pertaining to each analytical objective are summarized in Table 5 and the final revised version of PERCCI-S (PERCCI-S-Rev) in Table 6.

**Table 5:** PERCCI-S-Rev item development process.

Item tested	Modifications considered	Results and decision
2. They treat me with kindness, as though I matter to them. New wording: 2. The care workers treat me with compassion.	In the original validation, experts suggested emphasizing empathy or compassion instead of kindness as to better match the concept of PCC.	*Result:* The proportion of missing responses was reduced from 4.7% to 3.7%. The standardized factor loading was .88. Item response category characteristic curves and information curves provided more information. *Decision:* Replace item 2 with the reworded item.
3. They can tell my good days from my bad days. New wording: The care workers adapt the care to how I feel.	In the original validation, interviews with patients revealed that some had difficulties interpreting this item saying that they cannot know what the care workers think. The new phrasing was constructed trying to keep the original intention.	*Result:* The proportion of missing responses was reduced from 8.1% to 6.0%. The standardized factor loading was .89. Item response category characteristic curves and information curves provided more information. *Decision:* Replace item 3 with the reworded item.
5. They understand the areas of life I need help with. New wording: They understand what I need help with.	In the original validation, experts suggested removing the word life, arguing that it's not the HBPC's role to help the patient in every area of life.	*Result:* The proportion of missing responses was reduced from 7.1% to 4.2%. The standardized factor loading was .90. Item response category characteristic curves and information curves provided more information. *Decision:* Replace item 5 with the reworded item.
6. I am given enough time to say everything I want to say about my health and treatment. New wording: I am given enough time to say everything I want to say about my care.	Replacing “health and treatment” with “care” was considered as to emphasize the tasks of HBPC and not heath care in general.	*Result:* The proportion of missing responses was reduced from 5.6% to 3.5%. The standardized factor loading was greater than .89. Item response category characteristic curves and information curves provided more information. *Decision:* Replace item 6 with the reworded item.
7. My care and support helps me to be optimistic about what I can still do. New wording: The care I receive in my home helps me understand what I can do myself.	In the original validation, experts suggested rephrasing this item since the aim of the care is to support the patients’ independence, which is not the same as the patient feeling optimistic about what they still can do. The wording also gives the patient a passive role instead of an active partner.	*Result:* The proportion of missing responses was reduced from 7.7% to 4.4%. The standardized factor loading was .78. Item response category characteristic curves and information curves provided approximately the same amount of information. *Decision:* Keep the original item.
8. I have a say in decisions made about my care and support. New wording: I am involved in decisions about my home care to the extent that I want to be.	In the original validation, experts suggested rephrasing the item to better harmonize with modern concepts of PCC emphasizing a partnership between care workers and patients.	*Result:* The proportion of missing responses was reduced from 7.7% to 3.2%. The standardized factor loading was .86. Item response category characteristic curves and information curves provided more information. *Decision:* Replace item 8 with the reworded item.
9. The care workers help me coordinate my care. New item: The care workers have sufficient information about me and what needs to be done.	In the original validation, interviews with patients revealed that some patients did not need help coordinating their care, thus having trouble replying. During interviews conducted in the validation process of the original version, patients described that they regularly had to instruct the care workers what to do because they lacked necessary information. This dimension of PCC in HBPC was not covered in the original version.	*Result:* The proportion of missing responses was reduced from 7.3% to 3.8%. The standardized factor loading was .86. Item response category characteristic curves and information curves provided more information. *Decision:* Replace item 9 with the new item.
11. My opinions about my care and support are respected. New wording: My opinions about my home care are respected.	Replacing “care and support” with “home care” was considered in order to clarify that the item concerned HBPC and not to social services.	*Result:* The proportion of missing responses was reduced from 6.7% to 2.6%. The standardized factor loading was .93. Item response category characteristic curves and information curves provided more information. *Decision:* Replace item 11 with the reworded item.
12. My care and support strengthen my ability to manage my illness and treatment. New wording: The care I receive in my home strengthens my ability to manage my situation.	Replacing “care and support” with “home care” as well as “illness and treatment” with “situation” was considered in order to clarify that the item concerned HBPC and not to social services or other health care.	*Result:* The proportion of missing responses was reduced from 8.5% to 4.0%. The standardized factor loading was .86. Item response category characteristic curves and information curves provided approximately the same amount of information. *Decision:* Keep the original item.
New item 13. There are too many different care workers providing my home care.	In the original validation, interviews with patients highlighted that continuity of care is a key element of PCC. This aspect is not captured by existing PERCCI-S items.	*Result:* The standardized factor loading was .40. *Decision:* Item 13 is not added to the PERCCI-S-rev.
New item 14. It is difficult to get in contact with the home care when I need to.	In the original validation, interviews with patients revealed that access to care is a key element of PCC. This aspect is not captured by existing PERCCI-S items.	*Result:* The standardized factor loading was .43. *Decision:* Item 14 is not added to the PERCCI-S-rev.

**Table 6:** Different versions of PERCCI-S.

Original PERCCI-S	PERCCI-S-Rev (final version)
Response options (original): Never or rarely; Sometimes; Often; Always	Response options (revised): Never; Very seldom; Quite seldom; Sometimes; Quite often; Very often; Always
1. The care workers take what I say seriously.	1. The care workers take what I say seriously.
2. They treat me with kindness, as though I matter to them.	2. Revised: The care workers treat me with compassion.
3. They can tell my good days from my bad days.	3. Revised: They adapt the care to how I feel.
4. I have confidence in the care workers.	4. I have confidence in the care workers.
5. They understand the areas of life I need help with.	5. Revised: They understand what I need help with.
6. I am given enough time to say everything I want to say about my health and treatment.	6. Revised: I am given enough time to say everything I want to say about my care.
7. My care and support helps me to feel optimistic about what I can still do.	7. My care and support helps me to feel optimistic about what I can still do.
8. I have a say in decisions taken about my care and support.	8. Revised: I am involved in decisions about my home care to the extent that I want.
9. The care workers help me coordinate my care.	9. Revised: The care workers have sufficient information about me and what needs to be done.
10. I get help with the things that are most important to me.	10. I get help with the things that are most important to me.
11. My opinions about my care and support are respected.	11. Revised: My opinions about my home care are respected.
12. My care and support strengthen my ability to manage my illness and treatment.	12. My care and support strengthen my ability to manage my illness and treatment.

Items labelled “Revised” reflect wording changes relative to the original PERCCI-S; unchanged items were retained verbatim in the PERCCI-S-Rev.

## 4 Discussion

This study aimed to refine and validate a revised version of the Swedish version of the Person-Centred Community Care Inventory (PERCCI-S-Rev) for use in HBPC. As care provision increasingly shifts into patients' homes, and in line with the WHO's call for people-centred and integrated health systems, there is a growing need for instruments that accurately capture person-centredness from the patient's perspective. The main finding from this study is that the PERCCI-S-Rev demonstrates improved psychometric performance and greater clarity, including greater measurement precision and reduced ceiling effects, supporting its use for distinguishing across a wider range of patient-reported experiences. As such, it is well suited for routine monitoring of PCC in Swedish HBPC.

Existing instruments used to assess PCC often have notable limitations. Many lack grounding in an explicit conceptual framework of PCC, limiting the interpretability and relevance of the scores they generate (39, 40). As a result, the data produced often reflect the extent to which patients' experiences align with benchmarks defined by individuals other than the person receiving care (41). Some instruments have been developed based on theoretical frameworks to evaluate PCC from the patient's perspective. Most, however, are designed for use in specific contexts and patient groups, such as intermediate elderly care (between hospital and social care) (42), primary care (43, 44) and social services (45, 46). Understandably, instruments developed for other care settings cannot be used in HBPC without adaptation. For example, tools used in intermediate elderly care include items on discharge information (42), while many patients receiving HBPC in Sweden are never discharged, as this care is often provided until the end of life. Similarly, instruments developed for primary care include items relating to the patient's doctor (43), yet physicians are not employed within Swedish HBPC.

While several generic PCC instruments have been developed in recent years (47, 48), many are episode-based, multidimensional, or oriented toward clinical encounters rather than longitudinal home-based care. In contrast, PERCCI-S-Rev offers a brief, unidimensional measure suited for routine monitoring across municipal HBPC services.

The revised version enhances the original scale in several ways. By expanding the response options from four to seven, the PERCCI-S-Rev captures more nuanced differences in patients' experiences and reduces the ceiling effect that previously limited differentiation among highly satisfied respondents. The revised version provides its greatest measurement precision among respondents scoring at the lower end of the latent continuum, making it more informative among respondents reporting less favourable experiences—an important feature for identifying areas in need of improvement. Across analytic approaches, including IRT and EFA, the PERCCI-S-Rev demonstrated a coherent and unidimensional structure, supporting the use of a single composite score.

The revised scale demonstrated good internal consistency. Although high ordinal alpha values are sometimes interpreted as suggesting item redundancy or excessive scale length, decisions regarding item retention must also consider content validity and intended use. In the present case, the scale comprises 12 items designed to capture complementary aspects of the construct, and retaining this level of item coverage was considered important for quality-improvement applications, where attention is often directed toward identifying specific items with lower scores.

Rewording several items (2, 3, 5, 6, 8, 9 and 11) improved clarity, reduced missing responses, and strengthened factor loadings. For example, modifying items 2, 3, 5, and 6 led to lower nonresponse rates and stronger factor loadings, suggesting that subtle changes in wording can meaningfully improve measurement quality. Similarly, items 2, 3, 8 and 9 were rephrased to better align with contemporary concepts of person-centred care, emphasising patient involvement, respect, and autonomy, while also producing stronger psychometric performance.

Rewording item 7 to highlight patient independence led to weaker psychometric performance, and the original wording was therefore retained. Nonetheless, the item continues to pose conceptual challenges, and alternative phrasings should be explored in future validation studies. Potentially, framing the item around patients' capabilities (My care strengthens my ability to take care of my health) or autonomy (My care supports me in making my own decisions about my health) may provide a closer match to PCC principles. Such alternatives could first be tested qualitatively before large-scale validation.

The exploratory addition of two new items relating to continuity and access did not support extending the unidimensional scale. Although these domains are highly salient to patients and widely discussed as important aspects of care quality and person-centred service delivery (1, 49–53), they did not load coherently with the other 12 items when forming a single composite score. This suggests that continuity and access may represent a distinct dimension of the patient's experience. We therefore recommend retaining these items in the questionnaire to provide complementary information, while excluding them from the PERCCI-S-Rev scale score.

Because cooperation between health and social care is often central to achieving person-centredness (25, 27), stakeholders have questioned whether patient evaluations might be influenced by attitudes toward social care rather than HBPC itself. Although respondents were clearly instructed to evaluate experiences of municipal HBPC and explicitly asked to exclude social care services, separating these service domains may nevertheless be cognitively challenging for some patients, particularly for those receiving multiple forms of support in their home. In this context, measurement equivalence should be interpreted as an evaluation of whether the PERCCI-S-Rev functions comparably across patient groups with different care contexts, rather than as evidence that respondents fully separated evaluations of HBPC from social care in all cases. Measurement invariance analyses therefore served as psychometric safeguards to examine whether response patterns differed systematically between patients receiving HBPC alone and those receiving HBPC in combination with social care services. The results supported both metric and scalar invariance, indicating that the PERCCI-S-Rev functions equivalently across these care contexts. This supports the use of the overall PERCCI-S-Rev score in samples with combined HBPC and social care services.

### 4.1 Strengths and limitations

Strengths of this study include the large and diverse sample and the use of modern psychometric methods that are well suited to ordinal data and partially missing responses (54). Applying appropriate analytic techniques allowed us to make full use of the available data while maintaining methodological rigor. In addition, analyses of item non-response suggested that incomplete responses were to some extent related to respondent characteristics, including age and the need for assistance when completing the survey. This pattern highlights the value of using model-based methods and multiple imputation approaches when conducting psychometric analyses in the presence of missing item responses.

Several limitations should also be acknowledged. Although patients were engaged at several points in the process, they were not directly involved in evaluating the revised version. In addition, because the original PERCCI-S was developed in the context of a quality improvement project, opportunities for external convergent validation were limited by the availability of independent, conceptually related measures within the survey. Another important limitation is that we lacked data from municipal registers on whether patients had concurrent receipt of social care. Instead, this information was self-reported in the questionnaires. Four percent of respondents answered that they did not know, introducing potential misclassification. We were also unable to examine non-response patterns, so we could not assess whether groups such as people with cognitive impairments or those with a first language other than Swedish were less likely to participate, which could have led to their underrepresentation.

We also want to point out that although the present study provides a comprehensive evaluation of the revised instrument, a randomized or factorial design would be required to isolate the effects of individual modifications, such as item wording, response scale expansion, or the addition of new items. Studies using randomized allocation to alternative instrument versions are therefore warranted to directly compare performance under controlled conditions.

### 4.2 Future directions

Overall, the PERCCI-S-Rev appears to be a psychometrically robust and practically useful instrument for capturing patients’ perceptions of person-centredness in HBPC. It has potential applications in clinical practice, research, and policy—for benchmarking, monitoring trends, and guiding service improvement. Validation has so far been conducted only in Sweden, and further studies in other countries are needed to assess cross-cultural performance. As a next step, an argument-based approach to measurement validation is recommended to examine the scale's usefulness in evaluating interventions or service models aimed at strengthening person-centred care (55, 56).

Future research should prioritise external validation using independent measures of conceptually related constructs (e.g., involvement, respect, communication quality), as well as discriminant measures, to strengthen evidence for convergent and discriminant validity. In addition, future studies should examine measurement invariance and differential item functioning across key subgroups, such as age, gender, health status, and care contexts, using larger and more diverse samples to support the scale's use in comparative evaluations.

## 5 Conclusion

The revised PERCCI-S improves on the original with clearer wording and expanded response options that better distinguish patients' experiences. Supported by robust psychometric evidence and tested in a large and diverse patient population, the PERCCI-S-Rev is ready for implementation in Swedish HBPC settings, where it can be used to identify areas where person-centredness can be improved. We recommend that PERCCI-S-Rev is used instead of PERCCI-S.

## Data Availability

The datasets generated and/or analysed during the current study are not publicly available owing to confidentiality, but excerpts are available from the corresponding author upon reasonable request.
