# Supplementary material for: Development and validation of an enhanced PERCCI-S (PERCCI-S-Rev) for measuring person-centred home-based primary care
Source: Front Health Serv. 2026 Feb 27;6:1766556. doi: 10.3389/frhs.2026.1766556 (PMC12982167; doi:10.3389/frhs.2026.1766556)
Supplement: Supplementary file 1 [file Supplementaryfile1.docx]

**Comparison of response category characteristic curves and information curves for all tested items in the revised and original version of PERCCI-S**

**Item 1 response category characteristic curves**

**PERCCI-S-REV PERCCI-S**

The care workers take what I say seriously. The care workers take what I say seriously.

Probability

Probability

PERCCI-S score (Theta)

PERCCI-S score (Theta)

**Note:** The figures show the probability of selecting a particular response option where the value zero denotes the average and the value 1 denotes one standard deviation from the average.

**Item1 Information curves**

Item information

PERCCI-S score (Theta)

**Note:** Theta is the estimated standardized PERCCI-S score based on the IRT model, where the value zero denotes the average and the value 1 denotes one standard deviation from the average.

**Item 2 response category characteristic curves**

**PERCCI-S-REV PERCCI-S**

They treat me with compassion. They treat me with kindness, as though I matter to them.

Probability

Probability

PERCCI-S score (Theta)

PERCCI-S score (Theta)

**Note:** The figures show the probability of selecting a particular response option where the value zero denotes the average and the value 1 denotes one standard deviation from the average.

**Item 2 Information curves**

Item information

PERCCI-S score (Theta)

**Note:** Theta is the estimated standardized PERCCI-S score based on the IRT model, where the value zero denotes the average and the value 1 denotes one standard deviation from the average.

**Item 3 response category characteristic curves**

**PERCCI-S-REV PERCCI-S**

They adapt the care to how I am feeling. They can tell my good days from my bad days.

Probability

Probability

PERCCI-S score (Theta)

PERCCI-S score (Theta)

**Note:** The figures show the probability of selecting a particular response option where the value zero denotes the average and the value 1 denotes one standard deviation from the average.

**Item 3 Information curves**

Item information

PERCCI-S score (Theta)

**Note:** Theta is the estimated standardized PERCCI-S score based on the IRT model, where the value zero denotes the average and the value 1 denotes one standard deviation from the average.

**Item 4 response category characteristic curves**

**PERCCI-S-REV PERCCI-S**

I have confidence in the care workers. I have confidence in the care workers.

Probability

Probability

PERCCI-S score (Theta)

PERCCI-S score (Theta)

Note: The figures show the probability of selecting a particular response option where the value zero denotes the average and the value 1 denotes one standard deviation from the average.

**Item 4 Information curves**

Item information

PERCCI-S score (Theta)

**Note:** Theta is the estimated standardized PERCCI-S score based on the IRT model, where the value zero denotes the average and the value 1 denotes one standard deviation from the average.

**Item 5 response category characteristic curves**

**PERCCI-S-REV PERCCI-S**

They understand what I need help with. They understand the areas of life I need help with.

Probability

Probability

PERCCI-S score (Theta)

PERCCI-S score (Theta)

**Note:** The figures show the probability of selecting a particular response option where the value zero denotes the average and the value 1 denotes one standard deviation from the average.

**Item 5 Information curves**

Item information

PERCCI-S score (Theta)

**Note:** Theta is the estimated standardized PERCCI-S score based on the IRT model, where the value zero denotes the average and the value 1 denotes one standard deviation from the average.

**Item 6 response category characteristic curves**

**PERCCI-S-REV PERCCI-S**

I am given enough time to say everything I want to. I am given enough time to say everything I say about my home care. want to say about my health and care.

Probability

Probability

**Note:** The figures show the probability of selecting a particular response option where the value zero denotes the average and the value 1 denotes one standard deviation from the average.

**Item 6 Information curves**

Item information

PERCC-S score (Theta)

**Note:** Theta is the estimated standardized PERCCI-S score based on the IRT model, where the value zero denotes the average and the value 1 denotes one standard deviation from the average.

**Item 7 response category characteristic curves**

**PERCCI-S-REV PERCCI-S**The home care I receive helps me understand what I My care and support helps me to feel
can do myself. optimistic about what I can still do.

Probability

Probability

**Note:** The figures show the probability of selecting a particular response option where the value zero denotes the average and the value 1 denotes one standard deviation from the average.

**Item 7 information curves**

Item information

**Note:** Theta is the estimated standardized PERCCI-S score based on the IRT model, where the value zero denotes the average and the value 1 denotes one standard deviation from the average.

**Item 8 response category characteristic curves**

**PERCCI-S-REV PERCCI-S**

I am involved in decisions about my home care to the I have a say in decisions taken about my extent that I want. care and support.

Probability

Probability

PERCCI-S score (Theta)

PERCCI-S score (Theta)

**Note:** The figures show the probability of selecting a particular response option where the value zero denotes the average and the value 1 denotes one standard deviation from the average.

**Item 8 Information curves**

Item information

PERCCI-S score (Theta)

**Note:** Theta is the estimated standardized PERCCI-S score based on the IRT model, where the value zero denotes the average and the value 1 denotes one standard deviation from the average.

**Item 9 response category characteristic curves**

**PERCCI-S-REV PERCCI-S**

They have sufficient information about me and what The care workers help me coordinate my needs to be done. care.

Probability

Probability

PERCCI-S score (Theta)

PERCCI-S score (Theta)

**Note:** The figures show the probability of selecting a particular response option where the value zero denotes the average and the value 1 denotes one standard deviation from the average.

**Item 9 Information curves**

Item information

PERCCI-S score (Theta)

**Note:** Theta is the estimated standardized PERCCI-S score based on the IRT model, where the value zero denotes the average and the value 1 denotes one standard deviation from the average.

**Item 10 response category characteristic curves**

**PERCCI-S-REV PERCCI-S**

I get help with the things that are most important to me. I get help with the things that are most important to me.

Probability

Probability

PERCCI-S score (Theta)

PERCCI-S score (Theta)

**Note:** The figures show the probability of selecting a particular response option where the value zero denotes the average and the value 1 denotes one standard deviation from the average.

**Item 10 Information curves**

Item information

PERCCI-S score (Theta)

**Note:** Theta is the estimated standardized PERCCI-S score based on the IRT model, where the value zero denotes the average and the value 1 denotes one standard deviation from the average.

**Item 11 response category characteristic curves**

**PERCCI-S-REV PERCCI-S**My opinions about my home care are respected. My opinions about my care and support are
 respected.

Probability

Probability

**Note:** The figures show the probability of selecting a particular response option where the value zero denotes the average and the value 1 denotes one standard deviation from the average.

**Item 11 information curves**

Item information

PERCCI-S score (Theta)

**Note:** Theta is the estimated standardized PERCCI-S score based on the IRT model, where the value zero denotes the average and the value 1 denotes one standard deviation from the average.

**Item 12 response category characteristic curves**

**PERCCI-S-REV PERCCI-S**The home care I receive strengthens my ability to manage My care and support strengthens my ability my situation. to manage my illness and treatment.

Probability

Probability

PERCCI-S score (Theta)

PERCCI-S score (Theta)

**Note:** The figures show the probability of selecting a particular response option where the value zero denotes the average and the value 1 denotes one standard deviation from the average.

**Item 12 information curves**

Item information

PERCCI-S score (Theta)

**Note:** Theta is the estimated standardized PERCCI-S score based on the IRT model, where the value zero denotes the average and the value 1 denotes one standard deviation from the average.

**Item 13 response category characteristic curves**

There are too many different care workers providing my home care.

Probability

**Note:** The figures show the probability of selecting a particular response option where the value zero denotes the average and the value 1 denotes one standard deviation from the average.

**Item 13 information curve**

Item information

PERCCI-S score (Theta)

**Note:** Theta is the estimated standardized PERCCI-S score based on the IRT model, where the value zero denotes the average and the value 1 denotes one standard deviation from the average.

**Item14 Response category characteristic curves**

It is difficult to get in contact with the home care when I need to.

Probability

PERCCI-S score (Theta)

**Note:** The figures show the probability of selecting a particular response option where the value zero denotes the average and the value 1 denotes one standard deviation from the average.

**Item 14 Information curve**

Item information

PERCCI-S score (Theta)

**Note:** Theta is the estimated standardized PERCCI-S score based on the IRT model, where the value zero denotes the average and the value 1 denotes one standard deviation from the average.
